# 

**Published:** 1883-06

**Authors:** 


Vol. XXXVII. JUNE, 1883. No. 6.

### THE Dental Register.

A MONTHLY JOURNAL,

DEVOTED TO THE INTERESTS OF THE PROFESSION.

EDITED BY J. TAFT, D.D.S.

PUBLISHED BY

SPENCER & CROCKER,

OHIO DENTAL DEPOT,

Nos. 117, 119, 121 WEST FIFTH STREET, CINCINNATI, OHIO.

TERMS:—$2.00 Per Annum, in Advance. Single Copies, 25 cts.

